# Parenteral Nanoemulsions Loaded with Combined Immuno- and Chemo-Therapy for Melanoma Treatment

**DOI:** 10.3390/nano12234233

**Published:** 2022-11-28

**Authors:** Chiara Monge, Ian Stoppa, Chiara Ferraris, Annalisa Bozza, Luigi Battaglia, Luigi Cangemi, Gianluca Miglio, Stefania Pizzimenti, Nausicaa Clemente, Casimiro Luca Gigliotti, Elena Boggio, Umberto Dianzani, Chiara Dianzani

**Affiliations:** 1 Dipartimento di Scienza e Tecnologia del Farmaco, Università degli Studi di Torino, via Pietro Giuria 9, 10125 Torino, Italy; 2 Dipartimento di Scienze della Salute, Università del Piemonte Orientale, via Solaroli 17, 28100 Novara, Italy; 3 Dipartimento di Scienze Cliniche e Biologiche, Università degli Studi di Torino, Regione Gonzole 10, 10043 Orbassano, Italy; 4 Nanostructured Interfaces and Surfaces (NIS) Interdepartmental Centre, Università degli Studi di Torino, 10124 Torino, Italy; 5 Dipartimento di Scienze Cliniche e Biologiche, Università degli Studi di Torino, Corso Raffaello 30, 10124 Torino, Italy; 6 Azienda Ospedaliero-Universitaria Maggiore della Carità, Corso Giuseppe Mazzini 18, 28100 Novara, Italy

**Keywords:** Intralipid^®^, ICOS-Fc, combination therapy, melanoma

## Abstract

High-grade melanoma remains a major life-threatening illness despite the improvement in therapeutic control that has been achieved by means of targeted therapies and immunotherapies in recent years. This work presents a preclinical-level test of a multi-pronged approach that includes the loading of immunotherapeutic (ICOS-Fc), targeted (sorafenib), and chemotherapeutic (temozolomide) agents within Intralipid^®^, which is a biocompatible nanoemulsion with a long history of safe clinical use for total parenteral nutrition. This drug combination has been shown to inhibit tumor growth and angiogenesis with the involvement of the immune system, and a key role is played by ICOS-Fc. The inhibition of tumor growth in subcutaneous melanoma mouse models has been achieved using sub-therapeutic drug doses, which is most likely the result of the nanoemulsion’s targeting properties. If translated to the human setting, this approach should therefore allow therapeutic efficacy to be achieved without increasing the risk of toxic effects.

## 1. Introduction

Therapeutic options for melanoma depend upon disease staging. The surgical removal of a primary tumor is normally practiced in the case of early-stage disease (0–IIA). In stage IIB/C (tumor thickness > 2.0 mm) and stage III, adjuvant chemotherapies are practiced following surgery. Dacarbazine, the standard chemotherapy for stage IV (metastatic) melanoma up to 2011, is simply a palliative care treatment [1]. Temozolomide (TMZ) is an alternative treatment as it can reach the central nervous system (CNS) to treat brain metastases [2]. New pharmacological agents have recently been approved. Since half of the total melanomas show the V-raf murine sarcoma viral oncogene homolog B1 (BRAF) mutation, they may respond to targeted therapies with BRAF (vemurafenib, dabrafenib, encorafenib) and/or mitogen-activated protein kinase (MEK) inhibitors (trametinib, cobimetinib, binimetinib). Moreover, melanomas respond to immunotherapy with monoclonal antibodies that block the immune checkpoint receptors cytotoxic T-lymphocyte antigen 4 (CTLA4), such as ipilimumab, and programmed cell death protein 1 (PD1), such as pembrolizumab and nivolumab [1]. Indeed, melanoma is one of the most immunogenic tumors and its relationship with the host immune system is currently under investigation [3].

Besides immunotherapy with CTLA4 and PD1 inhibitors, we have recently reported the efficacy of targeting the inducible T-cell co-stimulator (ICOS)/ICOS ligand (ICOS-L) dyad in mouse models of melanoma [4]. ICOS [5,6,7] is an immune checkpoint protein, mainly present on activated T cells, that belongs to the cluster of differentiation 28 (CD28) family, with members such as CTLA4 and PD1. ICOS-L (or B7h) is its natural ligand, and is expressed by several cell types, such as B cells, macrophages, dendritic cells (DC), endothelial cells (EC), epithelial cells, fibroblasts, and several types of tumor cells. However, while CTLA4 and PD1 exert inhibitory functions on T cells, CD28 and ICOS trigger co-stimulatory signals. Indeed, the triggering of ICOS by ICOS-L co-stimulates T cells in inflamed tissues by modulating cytokine secretion from T helper cells, the cytotoxic activity of T cells, and the regulatory development of regulatory T cells [8,9]. On the other hand, the triggering of ICOS-L by ICOS inhibits the migration of vascular EC, DC, and tumor cells, as well as tumor metastatization in vivo [10,11]. In the tumor microenvironment, ICOS-L is expressed by several types of immune cells, by EC, and often by tumor cells, whereas ICOS is expressed by infiltrating T cells. We have recently found that the growth of established mouse melanoma is inhibited by treatment with ICOS-Fc, a recombinant water-soluble ICOS protein, when it is loaded into either biocompatible poly(lactic-co-glycolic acid) (PLGA) or cyclodextrin nanoparticles, which are able to target the drug to the tumor mass [4]. The main effect of ICOS-Fc is the inhibition of tumor angiogenesis, which is accompanied by variable immuno-regulatory activities, depending on the nanoparticle vector, and by direct effects on tumor cells, which are mainly detectable in vitro. Intriguingly, ICOS-L can also bind osteopontin (OPN), which is a well-known pro-neoplastic factor. Triggering of ICOS-L by OPN stimulates angiogenesis and tumor cell migration, whereas ICOS exerts a dominant negative effect on these activities [12]. Moreover, the ICOS/ICOS-L dyad may even play a role in anti-CTLA4 monoclonal antibody (mAb) anti-tumor activity, which results in an expansion of ICOS^+^ effector T cells, while its effect is significantly decreased in ICOS^-/-^ mice [13].

Nonetheless, despite significant therapeutic advancements, malignant melanoma remains an aggressive and resistant tumor with unpredictable responses to chemotherapy, making it a major health challenge [1]. Targeted therapies are hampered by chemoresistance [14], while the response to immunotherapy strictly correlates to tumor-mutation burden [15,16,17]. Therefore, a multi-pronged approach that can target melanoma proliferation, angiogenesis, and chemoresistance should be practiced, concurrently to immunotherapy, to improve therapeutic efficacy. We have recently shown that combinations of drugs against melanoma can be loaded into nanoemulsions for total parenteral nutrition, namely Intralipid^®^ 10% (IL), and that the combinations are effective in an in vivo mouse model of melanoma [18]. Specifically, a combination of drugs, including tumor proliferation (TMZ), angiogenesis (bevacizumab), and mTOR inhibitors (rapamycin), was loaded into IL and tested on available cell and animal models. Despite the promising results obtained, a strong dependence on rapamycin dose was observed as the relevant in vivo tumor inhibition was only achieved at a high dose of this drug. This poses a concern for human translation. Indeed, besides synergism between drugs, the rationale for anti-cancer drug combinations focuses on reducing the dose of each component to prevent side effects.

Hence, in the present work, we have optimized an innovative drug combination, which, after loading into IL, may be capable of acting at sub-therapeutic doses of each single component. To this aim, while TMZ was maintained as the main cytotoxic agent, the immune system was involved in the drug combination, unlike in our previous study, by including ICOS-Fc, capable to act on both the immune response and the tumor microenvironment. Since ICOS-Fc would not be compatible with the anti-angiogenic monoclonal antibody, bevacizumab, as they would exceed the maximum protein loading in the IL oil droplets, the new formulation included sorafenib (SOR), loaded into IL instead of rapamycin and bevacizumab, to counteract angiogenesis, which plays a key role in melanoma development. Indeed, SOR is a multi-kinase inhibitor that can exert anti-proliferative activity by inhibiting various intracellular, rapidly accelerated fibrosarcoma (RAF) kinases, including BRAF. As previously mentioned, targeted therapies for melanoma currently make use of selective BRAF inhibitors (such as vemurafenib), due to the specific melanoma mutational burden, but suffer from the significant limitation of chemoresistance. SOR displays broader anti-angiogenic activity than these compounds as it blocks the vascular endothelial growth factor receptor (VEGFR) with high affinity and at low therapeutic doses, as well as activating the immune response. It has also already been tested for melanoma treatment in combination with TMZ [19,20,21].

## 2. Experimental

### 2.1. Materials

#### 2.1.1. Chemicals

IL 10% was obtained from Fresenius Kabi (Bad Homberg, Germany). The 3-(4,5-dimethyl thiazol-2-yl)-2,5-diphenyltetrazolium bromide (MTT), 60,000–90,000 MW dextran, acetonitrile, crystal violet, dichloromethane, dimethylformamide (DMF), dimethylsulfoxide (DMSO), polystyrene sulfonate (PS), TMZ, and the TRIzol reagent were obtained from Sigma-Aldrich (St. Louis, MO, USA). Ethyl acetate, phosphotungstic acid, silica gel, and sodium docusate (AOT) were obtained from Merck (Darmstadt, Germany). Agarose CL 4B, isopropanol, triethylamine, and trifluoroacetic acid (TFA) were obtained from Alfa-Aesar (Haverhill, MA, USA). Acetic acid, bromododecane, and sodium nitrite were obtained from Carlo Erba (Val De Reuil, France). SOR was obtained from LcLabs (Woburn, MA, USA). Sulfuric acid was obtained from Fluka (Buchs, Switzerland). ICOS-Fc was obtained from Novaicos (Novara, Italy). Kolliphor^®^ EL was a kind gift from BASF (Ludwigshafen am Rhein, Germany).

Matrigel was obtained from BD Biosciences (San Jose, CA, USA). Polyclonal rabbit anti-cluster of differentiation 31 (CD31) was obtained from Abcam (Cambridge, UK). Monoclonal mouse anti-human Kiel original clone 67 (Ki-67) antigen was obtained from Thermo Fisher Scientific (Waltham, MA, USA). The QuantiTect Reverse Transcription Kit was obtained from Qiagen (Hilden, Germany). The TaqMan gene expression Assay-on-Demand and TaqMan Universal PCR Master Mix were obtained from Applied Biosystems (Foster City, CA, USA). The CFX96 System was obtained from Bio-Rad Laboratories (Hercules, CA, USA).

#### 2.1.2. Cells

A2058 and M14 human melanoma cells and B16-F10 murine melanoma cells were purchased from the American Type Culture Collection (ATCC; Manassas, VA, USA). D4M cells, a mouse melanoma engineered cell line harboring the BRAFV600E mutation, were a gift from D.W. Mullis, Department of Medicine, Norris Cotton Cancer Center, Geisel School of Medicine at Dartmouth, Lebanon, NH, USA. M14 and B16-F10 cells were cultured in Roswell Park Memorial Institute 1640 medium (RPMI 1640; Sigma-Aldrich, St. Louis, MO, USA), while A2058 and D4M were cultured in Dulbecco’s Modified Eagle Medium (DMEM; Sigma-Aldrich, St. Louis, MO, USA). All culture media were supplemented with 10% fetal calf serum (FCS; PAA Laboratories, Pasching, Austria), penicillin/streptomycin (100 units/mL), and L-glutamine (2 mmol/L) (both from Sigma-Aldrich, St. Louis, MO, USA). Cell lines were cultured in a 5% CO_2_, 37 °C incubator.

#### 2.1.3. Animals

C57BL/6J mice were obtained from The Jackson Laboratory (Bar Harbor, ME, USA) and were then bred under pathogen-free conditions in the animal facility of the University of Piemonte Orientale, Department of Health Sciences (Authorization No. 61/2005-A—6 May 2005, issued by the General Directorate of Veterinary and Food Health—Italian Ministry of Health). Experiments were performed using female, 6-to-9-week-old C57BL/6J mice that were treated in accordance with the University Ethical Committee and European guidelines (Experimental protocol authorization No. 241/2022-PR, released on the 15-04-2022 by the Italian Ministry of Health for protocol No. DB064.76).

### 2.2. Preparation of Formulations under Study

#### 2.2.1. Prodrug Synthesis

A lipophilic TMZ dodecyl ester (TMZ-C12) was synthesized, in accordance with the literature [18,22,23].

#### 2.2.2. Formulation of Drug Combination-Loaded IL

**Figure 1:**
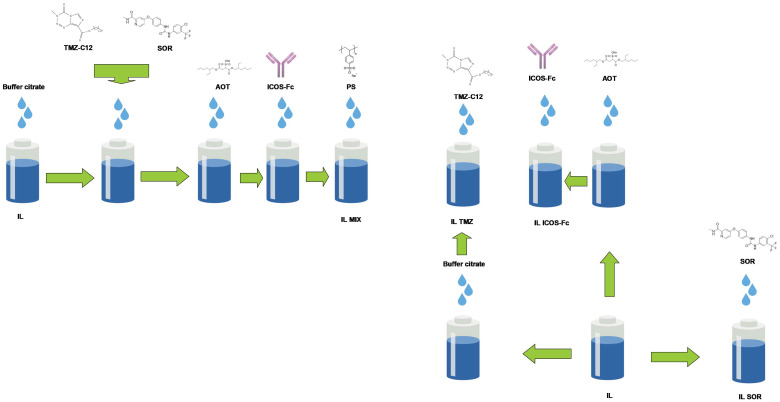
Flowchart of the preparation of the drug-loaded nanoemulsions. Abbreviations: AOT: sodium docusate; IL: Intralipid^®^ 10%; SOR: sorafenib; TMZ-C12: temozolomide dodecyl ester; MIX: drug combination (temozolomide dodecyl ester, sorafenib, ICOS-Fc); PS: polystyrene sulfonate.

The combination of drugs (IL MIX) was loaded into IL 10% in accordance with the following procedure. pH = 3.0 buffer citrate 0.1 M (40 μL) was first added to 1.6 mL of IL. Then, 1.2 mg of TMZ-C12 was dissolved in 80 μL of DMF, together with 0.5 mg of SOR, and this solution was added dropwise to IL. Subsequently, 96 uL of the AOT stock solution (4.5 mg/mL—10.1 mM) and variable amounts of the ICOS-Fc stock solutions (either 286 μL of 1.75 mg/mL human ICOS-Fc, or 302 μL of 1.65 mg/mL mouse ICOS-Fc) were added to IL, forming an ion pair [18,24] at a 1:150 AOT-ICOS-Fc molar ratio. The final drug concentrations in IL were: 0.6 mg/mL (1.65 mM) TMZ-C12, 0.25 mg/mL (0.54 mM) SOR, and 0.25 mg/mL (3.2 μM) ICOS-Fc. PS was used as the de-bridging agent exclusively in the case of the formulation of IL MIX with human ICOS-Fc. To this aim, 100 μL of a 10 mg/mL PS solution in water was added to avoid droplet aggregation (Figure 1). Human ICOS-Fc was used for in vitro studies on human cell lines (M14, A2058). Mouse ICOS-Fc was used for in vitro studies on mouse cell lines (B16, D4M) and for animal experiments.

#### 2.2.3. Preparation of Control Formulations

The following IL-based controls were used for cell studies: IL TMZ-C12, IL SOR, and IL ICOS-Fc (Figure 1). IL ICOS-Fc was also prepared using a 10-fold lower ICOS-Fc dose (0.025 mg/mL—0.32 μM) for use exclusively in in vitro cell migration experiments. The free-drug solution controls were: free TMZ, dissolved in DMF (6 mg/mL—31.2 mM), free SOR, dissolved in DMSO (5 mg/mL—10.8 mM), and free ICOS-Fc mg/mL, dissolved in water (1.75 mg/mL—22.4 μM for human ICOS-Fc; 1.65 mg/mL—22.1 μM for murine ICOS-Fc). A mixture of the free drugs (MIX) was obtained impromptu from the single stock solutions.

The free MIX for the animal experiments was prepared as follows: SOR (0.25 mg/mL—0.54 mM) was dissolved in Kolliphor^®^ EL/ethanol/normal saline (1:1:6 volume ratio), with mouse ICOS-Fc being added to a final concentration of 0.25 mg/mL (3.2 μM) and TMZ powder being added to the formulation prior to use (0.32 mg/mL—1.65 μM) in order to avoid pH-dependent degradation.

### 2.3. Characterization of Formulations

#### 2.3.1. Determination of Droplet Size, Morphology, and Zeta Potential

The dynamic light scattering technique (DLS; 90 Plus, Brookhaven, NY, USA) was used to determine the mean droplet size, polydispersity index (PDI), and Zeta potential of the IL-based formulations, at 25 °C and in triplicate. Measurement angles were 90° for particle size and 15° for Zeta potential. Transmission electron microscopy (TEM; High-Resolution JEOL 300 kV) was used via IL-negative staining with 1% phosphotungstic acid [18,25].

#### 2.3.2. Determination of Drug Recovery and Entrapment Efficiency

Drug recovery, defined as the ratio between the actual and theoretical drug concentrations, was determined by high-pressure liquid chromatography (HPLC) [18]. TMZ-C12 and SOR were extracted via the dilution of 50 μL of IL-based formulations in 100 μL of acetonitrile under a vortex, and centrifuging at 14,000 rpm (Allegra 64R centrifuge, Beckman Coulter, Brea, CA, USA). To extract the ICOS-Fc–AOT ion pair, the precipitate obtained in the previous step was dissolved in 100 μL of acetic acid, and lipids were precipitated with 50 μL of water (14,000 rpm centrifugation). Since PS interferes with the HPLC detection of ICOS-Fc, the recovery of IL MIX that was formulated with human ICOS-Fc was determined prior to its addition as the de-bridging agent. Drug entrapment efficiency (EE%), defined as the ratio between the drug amount entrapped in the lipid matrix and the total drug amount in the nanoemulsion, was assessed for each single therapeutic agent either after size exclusion with Agarose CL 4B, or after gradient centrifugation with 30% 60,000–90,000 MW dextran.

#### 2.3.3. HPLC Analysis of MIX

HPLC analyses were performed by modifying a literature method [18,26]. The HPLC system was composed of a YL9110 quaternary pump, a YL9101 vacuum degasser, and a YL9160 photo diode array (PDA) detector, linked to YL-Clarity software for data analysis (Young Lin, Hogyedong, Anyang, Korea). The column was a 300 nm pore size C8 Tracer Excel, 25 × 0.4 cm (Teknokroma, Barcelona, Spain). A gradient was performed at 75 °C and using a 1 mL/min flow rate between eluent A (0.1% TFA) and eluent B (79% isopropanol, 20% acetonitrile, 10% water, 0.1% TFA): 0 min–90% A; 15 min–40% A; 24 min–40% A; 27 min–90% A. The PDA wavelengths were 220 nm (ICOS-Fc), 265 nm (SOR), and 329 nm (TMZ-C12), and the retention times were 11.3 min for ICOS-Fc, 16.2 min for SOR, and 20.6 min for TMZ-C12.

### 2.4. Cytotoxicity: MTT Assay

Cells (1 × 10^3^/well) were seeded in 96-well plates for 24 h and then treated with the formulations under study. Viability was assessed via an MTT assay at 72 h, according to the manufacturer’s instructions. Four replicates were performed in five separate experiments.

### 2.5. Proliferation: Clonogenic Assay

The B16, D4M, M14, and A2058 melanoma cell lines (8 × 10^2^/well) were seeded into six-well plates. After 24 h, cells were treated with the formulations under study for 3 h. Afterwards, the medium was changed, and cells were cultured in drug-free medium for an additional 7 days. The clonogenic assay was then performed as previously described [18].

### 2.6. Invasion: Boyden Chamber Assay

Preliminary experiments were performed to identify non-toxic drug concentrations. B16, D4M, M14, and A2058 melanoma cells (8 × 10^3^) were seeded into 96-well plates and treated for 6 h with the formulations under study. Cell viability was assessed using the Crystal Violet assay, as previously described [18]. Melanoma cells (2 × 10^3^) were then plated onto the apical side of a Boyden chamber with filters (0.5 μm pore size and 8.2 mm diameter) that were coated with 50 μg/mL of Matrigel in serum-free medium. The cells were then either treated with non-toxic concentrations (as previously assessed) of the formulations under study or left untreated. The Boyden chamber-invasion assay was performed as previously described [18].

### 2.7. SOR Release from IL and Internalization into Melanoma Cells

#### 2.7.1. SOR Release from IL

Here, 1 mL of IL SOR was diluted in 4 mL of RPMI under magnetic stirring. At scheduled times, 0.5 mL of the mixture was withdrawn and centrifuged at 25,000 rpm (Allegra 64R centrifuge, Beckman Coulter, Brea, CA, USA), and the obtained supernatant was injected into the HPLC system. The SOR amount that was still present in the lipid matrix at the end of the experiment was assessed via extraction from the centrifuged lipid pellet. Briefly, the pellet was dissolved in 0.5 mL of acetonitrile and the lipid was precipitated with 0.5 mL of water, followed by centrifugation at 25,000 rpm (Allegra 64R centrifuge, Beckman Coulter, Brea, CA, USA).

#### 2.7.2. SOR Internalization in Melanoma Cells

Briefly, 1 μL of free SOR and, separately, 20 μL of IL SOR were diluted in 1 mL of RPMI, with and without FCS, containing 5 × 10^3^ melanoma cells. After 3 h of incubation, the cells were isolated by centrifugation and the pellet obtained was extracted using 50 μL of methanol, prior to injection into the HPLC system.

#### 2.7.3. HPLC Analysis of SOR

A Jasco PU 1580 pump and a C18 Beckmann ODS 25 × 0.5 cm column were used. The mobile phase, acetonitrile/water 65:35, was delivered at a flow rate of 1 mL/min. The Jasco UV 1575 UV-visible detector was set at λ 264 nm, and the calibration curve ranged between 2.5 and 0.25 ug/mL (R^2^ = 0.9994, CV = 0.070, LOD = 2.80 ng/mL, LOQ = 9.34 ng/mL).

### 2.8. Animal Experiments

B16-F10 melanoma cells were injected subcutaneously (1 × 10^5^ in 100 μL/mouse), and tumor growth was monitored every two days. Ten days after tumor induction, the mice were divided into different groups (five mice each; T0) and either treated, via i.v. injection, with the formulations under study, or with the same volume of phosphate-buffered saline (PBS), used as a control. Mice were treated three times a week for two weeks (six i.v./mouse, T1 to T6) and sacrificed three days after the last injection (Tend), or immediately after they displayed suffering. In each treatment, drug doses were: TMZ 1.5 mg/kg, SOR 1.25 mg/kg, and ICOS-Fc 1.25 mg/kg. Tumor volume was monitored over the treatment period and the final tumor mass and volume were measured at the end of the experiment, after animal sacrifice.

### 2.9. Immunohistochemistry of Tumor Specimens

The immunohistochemical analyses of CD31, an EC marker used to assess tumor micro-vessel density (TMD), and Ki-67, a marker of proliferating cells, were performed in animal-tumor specimens, as previously described [18].

### 2.10. Real-Time Polymerase Chain Reaction (PCR) of Tumors

Ribonucleic acid (RNA) was obtained from snap-frozen tumors, using the TRIzol reagent. Then, 1 μg of RNA was retrotranscribed to cDNA using the QuantiTect Reverse Transcription Kit. Interferon-γ (IFN-γ), interleukin-1β (IL-1β), interleukin-6 (IL-6), interleukin-10 (IL-10), and tumor necrosis factor-α (TNF-α) expression were evaluated using a TaqMan gene expression assay. The complementary deoxyribonucleic acid (cDNA) amounts were normalized using the β-actin gene. Real-time PCR was performed on a CFX96 System and samples were run in duplicate in a 10 μL final volume that contained 1 μL of diluted cDNA, 5 μL of TaqMan Universal PCR Master Mix, and 0.5 μL of Assay-on-Demand mix. Relative gene expression was calculated using the ΔΔ threshold cycle method.

### 2.11. Statistical Analysis

Data are presented as mean ± SEM. Statistical analyses were performed using Prism 3.0 software (GraphPad Software, La Jolla, CA, USA) by means of one-way ANOVA and the Dunnett’s test.

## 3. Results

### 3.1. Characterization of Formulations

Table 1 shows the characterization of the IL-based formulations. TMZ was loaded into the lipid matrix by means of its ester prodrug, which also increases its stability in biological fluids, preventing premature imidazotetrazine ring-opening at neutral pH [18,27]. ICOS-Fc is a high-molecular-weight and hydrophilic protein that was associated with the lipid matrix via ion pairing with AOT [18,24]. However, in some cases, the high density of positively charged amino groups on the ICOS-Fc molecule can cause the negatively charged IL droplets to aggregate, especially when the combination of drugs is loaded together with ICOS-Fc in the lipid matrix, meaning that PS was added, as a de-bridging agent, to the IL MIX formulation with human ICOS-Fc.

Overall, the drug combination was efficiently loaded into the lipid matrix without relevant changes in mean IL droplet size. The EE% of ICOS-Fc was determined exclusively on IL ICOS-Fc in the absence of PS, which would prevent the HPLC detection of the protein, as previously reported. The loading of SOR, whether alone or used in combination, led to a reduction in the Zeta potential absolute value, and this is probably due to the amino group present in the compound. The same occurred with ICOS-Fc, and this is probably caused by the excess amino groups of the protein that are exposed on the IL surface. However, the original Zeta potential was restored when the negatively charged PS was added as a de-bridging agent.

**Figure 2:**
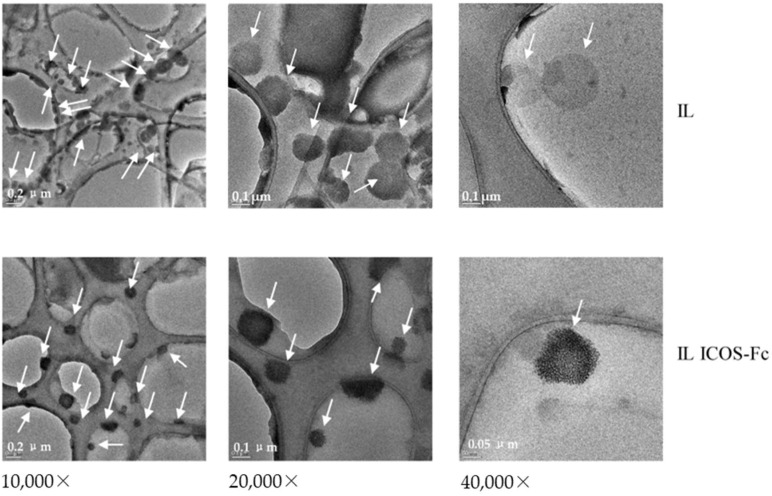
Transmission electron microscopy (TEM) images of Intralipid^®^ (IL) and ICOS-Fc-loaded IL.

The loading of ICOS-Fc was further investigated by TEM (Figure 2). The presence of condensed material on the surface of the IL droplets may be attributed to the ion-paired protein that is loaded into IL, as shown in a previous work by our research group [18].

### 3.2. In Vitro Studies

**Figure 3:**
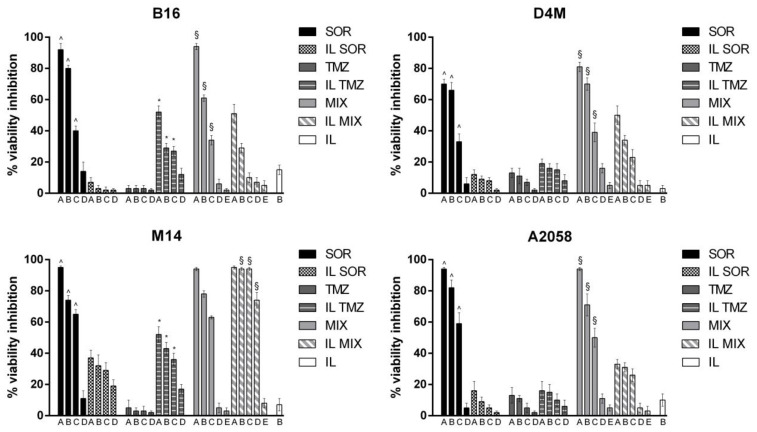
MTT assay performed after 72 h of incubation with the formulations under study. B16 cells: upper left panel; D4M cells: upper right panel; M14 cells: lower left panel; A2058 cells: lower right panel. Abbreviations: IL: Intralipid^®^ 10%; SOR: sorafenib; TMZ: temozolomide; MIX: drug combination (temozolomide dodecyl ester, sorafenib, ICOS-Fc). Concentrations employed: SOR A = 16 μM; B = 10 μM; C = 8 μM; D = 4.5 μM; E = 0.8 μM. TMZ A = 48 μM; B = 32 μM; C = 24 μM; D = 16 μM; E = 2.4 μM. Statistical analysis: ^ *p* < 0.05 SOR vs. IL SOR; * *p* < 0.05 TMZ vs. IL TMZ; § *p* < 0.05 MIX vs. IL MIX.

**Figure 4:**
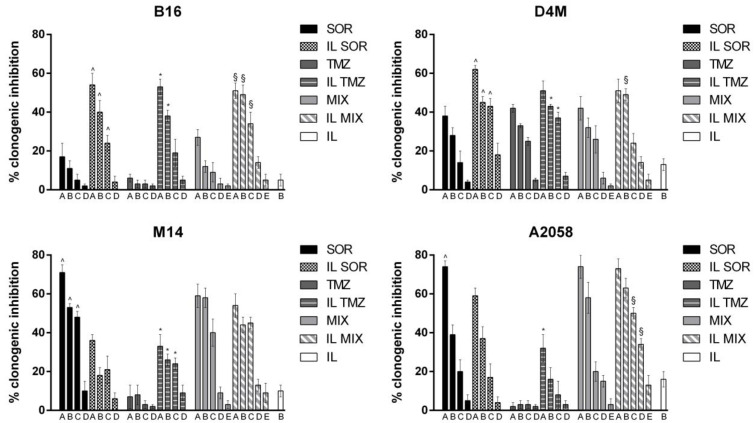
Clonogenic assay with B16 (upper left panel), D4M (upper right panel), M14 (lower left panel), and A2058 (lower right panel) melanoma cells. Abbreviations: IL: Intralipid^®^ 10%; SOR: sorafenib; TMZ: temozolomide; MIX: drug combination (temozolomide dodecyl ester, sorafenib, ICOS-Fc). Cells were treated with the formulations under study for 3 h. Afterwards, the medium was changed, and cells were cultured in drug-free medium for an additional 7 days. Concentrations employed: SOR A = 16 μM; B = 10 μM; C = 8 μM; D = 4.5 μM; E = 0.8 μM. TMZ A = 48 μM; B = 32 μM; C = 24 μM; D = 16 μM; E = 2.4 μM. Statistical analysis: ^ *p* < 0.05 SOR vs. IL SOR; * *p* < 0.05 TMZ vs. IL TMZ; § *p* < 0.05 MIX vs. IL MIX.

**Figure 5:**
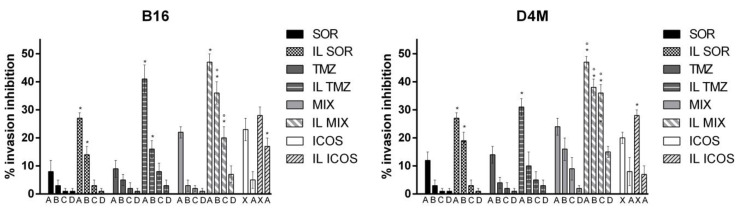
Migration assay with B16 (left panel) and D4M (right panel) melanoma cells. Abbreviations: IL: Intralipid^®^ 10%; SOR: sorafenib; TMZ: temozolomide; MIX: drug combination (temozolomide dodecyl ester, sorafenib, ICOS-Fc). Concentrations employed: SOR A = 16 μM; B = 10 μM; C = 8 μM; D = 4.5 μM. TMZ A = 48 μM; B = 32 μM; C = 24 μM; D = 16 μM. ICOS X = 2 μg/mL; A = 0.5 μg/mL. Statistical analysis: * *p* < 0.05 IL-loaded vs. free; ° *p* < 0.05 additive effect between drugs.

To preliminarily assess the biological effects of the IL formulations, we evaluated the effect on cell viability assessed by the MTT (Figure 3) and clonogenic (Figure 4) assays using the B16, D4M, M14, and A2058 cell lines, and on cell invasion (Figure 5) using B16 and D4M cells. The ICOS-Fc control (low concentration) was only included in the invasion experiments (Figure 5) since it is known not to affect cell viability (MTT—Figure 3, clonogenic assay—Figure 4).

The comparison between the activity of free drugs and that of the corresponding IL-loaded ones showed that IL loading always increased the inhibitory effect on cell invasion. In contrast, the effect on cell toxicity (i.e., inhibition of cell viability) was variable using the different drugs, cell lines, and assays. Compared to free drug, IL SOR decreased the cell toxicity detected by MTT in all the cell lines, whereas in B16 and D4M (mouse cell lines), it increased that detected by the clonogenic assay. IL loading increased TMZ cell toxicity in all the cell lines, even if in D4M and A2058 a less pronounced effect was detected by the MTT compared to the clonogenic assay. IL loading of MIX increased the cell toxicity detected by the clonogenic assay in all the cell lines, except in M14, where it showed no effect; while, with MTT, it increased cell toxicity only in M14, displaying the opposite effect in the other cells. In the MTT assay, an additive effect between IL SOR and IL TMZ could be hypothesized for IL MIX on M14 cells, since this is the only cell line where IL SOR exerts a relevant cytotoxic effect.

**Figure 6:**
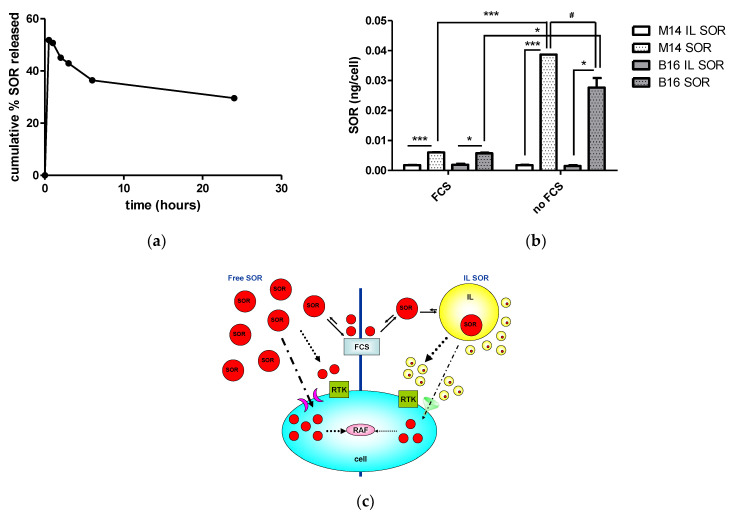
Hypothesized sorafenib (SOR) mechanism of action: (**a**) SOR release profile in culture medium, and (**b**) SOR internalization migration assay (B16, M14 melanoma cells): *** *p* < 0.005, * *p* < 0.05, # *p* < 0.01. (**c**) Hypothesized cellular pathways. Abbreviations: FCS: fetal calf serum; IL: Intralipid^®^ 10%; RAF: rapidly accelerated fibrosarcoma kinases; RTK: receptor tyrosine kinases.

Considering that interpreting the effect of IL MIX is complex, because it is influenced by the single drugs and the carrier, besides the cell phenotype, the most controversial results came from SOR-based formulations. This evidence further drove our investigations into the SOR mechanism of action. Release experiments in cell culture medium showed the unexpected profile that is depicted in Figure 6a. After an initial burst release, drug concentration decreased over time in the release medium. This cannot be ascribed to drug degradation, as the compound that was missing from the release medium was recovered in the lipid pellet obtained after centrifugation. Indeed, it appears that competition occurs between the release medium and the lipid matrix of IL. The internalization studies in melanoma cell lines (Figure 6b) showed that FCS strongly inhibited the internalization of free SOR, while it was ineffective on IL SOR. Moreover, IL SOR internalization was lower than that of free SOR, and this is probably because IL has an entry mechanism that is subject to saturation.

The mechanism depicted in Figure 6c may therefore be hypothesized. IL SOR internalization might be limited by a saturation-like effect, thus reducing the total SOR internalized within the cell, leading to reduced cytotoxic action (MTT assay), that is mediated exclusively by the inhibition of intracellular RAF kinases. On the other hand, extracellular SOR acts on receptor tyrosine kinases (RTK), which are located on the cell membrane and are responsible for angiogenesis and migration processes [28]. In the case of cell membrane-associated RTK, the availability of extracellular SOR in the culture medium is lowered by interactions with proteins, such as those of FCS [29,30]. When SOR is loaded into IL, competition is established between the lipid matrix and the culture medium, preventing SOR from protein sequestration and inactivation effects, thus resulting in more pronounced migration inhibition. The clonogenic assay, instead, entails the proliferation process, which is regulated, to various extents, by both RAF kinases and RTK, and this probably accounts for the variable results obtained among cell lines. In this case, the most striking differences were found between mouse (B16 and D4M) and human (M14 and A2058) cell lines, whose cellular targets (that is RAF kinases and RTK) could probably show different sensitivities to SOR.

These considerations suggest that the variable results obtained in the cell experiments with IL SOR may be ascribable to the experimental setting, rather than to the formulation itself.

### 3.3. In Vivo Studies

Animal experiments were performed by comparing the growth inhibition of IL MIX and free MIX on the established subcutaneous B16-F10 melanoma mouse model. Moreover, an animal group treated with IL ICOS-Fc was also included to clarify the contribution of immunotherapy to the total therapeutic effect.

**Figure 7:**
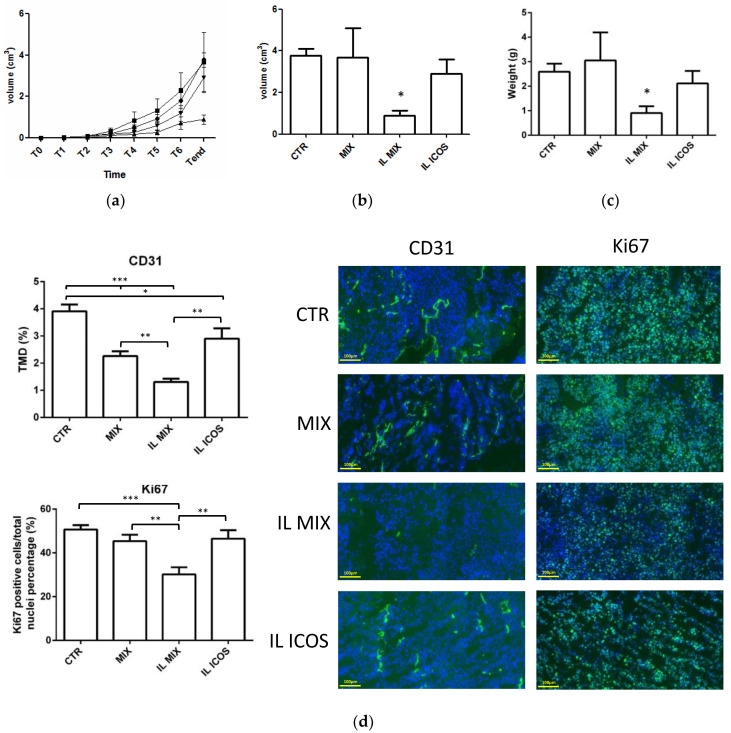

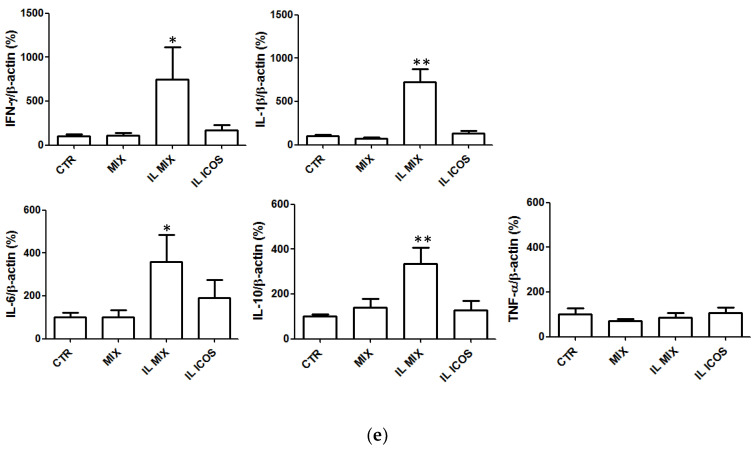
Animal experiments (I): (**a**) time course tumor volume, (**b**) endpoint tumor volume, (**c**) endpoint tumor weight, (**d**) tumor immunohistochemistry, and (**e**) tumor cytokines. Abbreviations: CD31: cluster of differentiation 31; CTR: control; IFN-γ: Interferon-γ; IL: Intralipid^®^ 10%; IL-10: Interleukin-10; IL-1β: Interleukin-1β; IL-6: Interleukin-6; IL ICOS: Intralipid^®^ 10% loaded with ICOS-Fc; IL MIX: Intralipid^®^ 10% loaded with temozolomide dodecyl ester, sorafenib, and ICOS-Fc; Ki-67: Kiel original clone 67; MIX: free drug combination (temozolomide dodecyl ester, sorafenib, ICOS-Fc); TMD: tumor micro-vessel density; TNF-α: tumor necrosis factor-α. Days after B16-F10 cell subcutaneous injection (1 × 10 ^5^ in 100 μL/mouse): T0 = 10 days; T1 = 14 days; T2 = 16 days; T3 = 19 days; T4 = 21 days; T5 = 23 days; T6 = 25 days; Tend = 28 days. T0: assignment to groups; T1 to T6: treatments; Tend: sacrifice. For this experiment, 20 mice were used (*n* = 5 each group). Statistical analysis *** = *p* < 0.0001; ** *p* < 0.005; * *p* < 0.05.

The results (Figure 7) show that only IL MIX was able to significantly reduce tumor volume and the mitotic index (Ki67), compared to the control animals. In contrast, tumor angiogenesis (CD31) was also decreased in animals treated with IL ICOS-Fc. Moreover, only treatment with IL MIX altered the cytokine expression pattern, inducing significant increases in IFN-γ, IL-1β, IL-6, and IL-10, while no effect on TNF-α was measured. No substantial toxicity was detected in analyses of the target organs, except for a slight increase in spleen weights for all the treated groups (Table 2).

**Figure 8:**
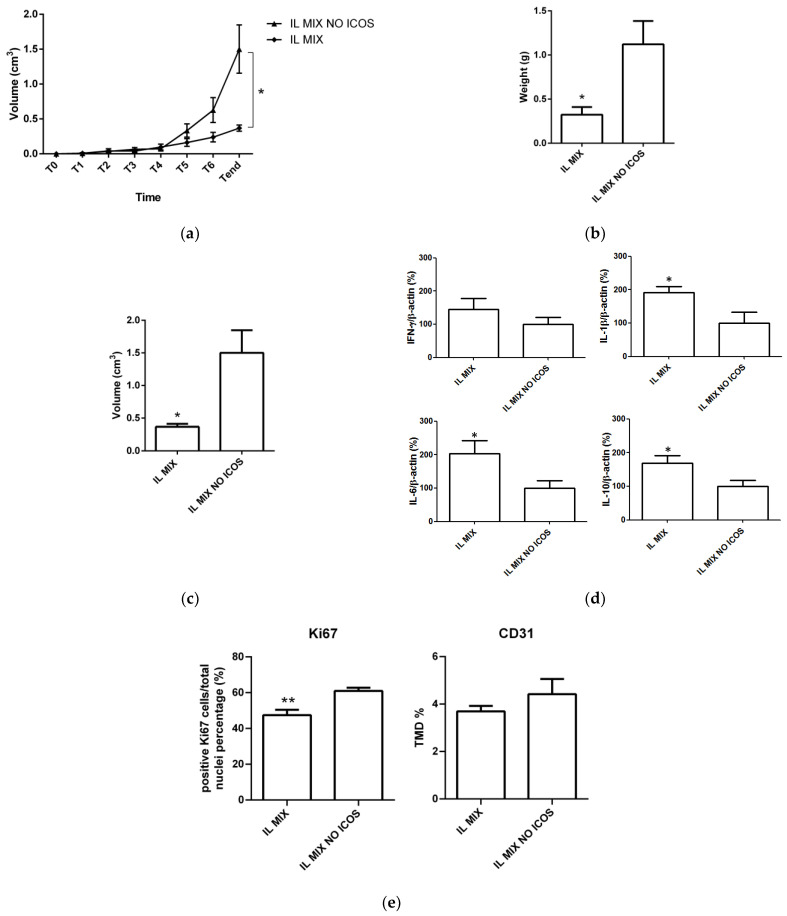
Animal experiments (II): (**a**) time-course tumor volume, (**b**) endpoint tumor volume, (**c**) endpoint tumor weight, (**d**) tumor cytokines, and (**e**) tumor immunohistochemistry. Abbreviations: IL: Intralipid^®^ 10%; CD31: cluster of differentiation 31; IFN-γ: Interferon-γ; IL-10: Interleukin-10; IL-1β: Interleukin-1β; IL-6: Interleukin-6; IL MIX: Intralipid^®^ 10% loaded with temozolomide dodecyl ester, sorafenib, and ICOS-Fc; IL MIX NO ICOS: Intralipid^®^ 10% loaded with temozolomide dodecyl ester and sorafenib; Ki-67: Kiel original clone 67; TMD: tumor micro-vessel density. Days after B16-F10 cell subcutaneous injection (1 × 10 ^5^ in 100 μL/mouse): T0 = 10 days; T1 = 14 days; T2 = 16 days; T3 = 19 days; T4 = 21 days; T5 = 23 days; T6 = 25 days; Tend = 28 days. T0: assignment to groups; T1 to T6: treatment; Tend: sacrifice. For this experiment, 10 mice were used (*n* = 5 each group). Statistical analysis ** *p* < 0.005; * *p* < 0.05.

Further in vivo experiments were performed to investigate the therapeutic contribution of ICOS-Fc in the IL MIX, and to this aim, the effect of IL MIX was compared to that of IL MIX formulated without ICOS-Fc (Figure 8, Table 3). The removal of ICOS-Fc from IL MIX resulted in it having a lesser effect on tumor growth (mass, volume), cell proliferation (Ki67), and immune modulation, in terms of IL-1β, IL-6, and IL-10 expression. In contrast, no significant differences were detected in terms of angiogenesis (CD31) and IFN-γ expression.

## 4. Discussion

### 4.1. Challenges of Current Melanoma Chemotherapy

Targeted therapies and immunotherapies have allowed the improved therapeutic control of malignant melanoma to be achieved. However, several drawbacks (e.g., chemoresistance for targeted therapies, need for high mutational burden in immunotherapies) still limit the effectiveness of these approaches. It is worth noting that combinations of the two are currently under study and have provided promising results, despite the higher incidence of side effects [1]. This experimental study therefore proposes a multi-target approach that merges immunotherapy (ICOS-Fc), targeted therapy (SOR), and chemotherapy (TMZ) and evaluates it at a preclinical level. This approach targets three of the major factors driving melanoma growth, i.e., proliferation, angiogenesis, and the immune response. Using previous encouraging results that have been achieved by our research group [18], this combination was loaded into a biocompatible colloidal vehicle, namely the nanoemulsion for total parenteral nutrition. This formulation is already employed in marketed drug-delivery systems and is under evaluation for anti-cancer drug delivery because of its range of potential targeting mechanisms [31]. Indeed, passive targeting mechanisms are favored by both its nanometric size range and its high lipid content, which acts by saturating the reticuloendothelial system (RES) [32]. Moreover, recent findings showed that it can reduce blood viscosity, by interrupting the binding between fibrinogen and red blood cells, and thus increase the tumor blood flow, which plays a key role in passive targeting. Indeed, upregulation of fibrinogen has been reported in cancer, whereas fibrinogen-mediated clot formation is responsible for the reduced tumor blood flow, a major barrier to drug delivery to tumors [33].

### 4.2. Advantages of Merging Different Approches into One Biocompatible Lipid Vehicle

The variability of the data obtained, especially on cell toxicity in the case of SOR, is mainly a result of the experimental in vitro setting, which does not account for the in vivo fate of drug-loaded IL. Indeed, the in vivo results indicate that IL MIX has promising therapeutic efficacy, while no relevant signs of toxicity were detected due to the sub-therapeutic doses employed for each compound: in our experiments, TMZ 1.5 mg/kg, SOR 1.25 mg/kg, and ICOS-Fc 1.25 mg/kg were co-administered, while in the literature TMZ 40.0 mg/kg, SOR 9.0 mg/kg, and ICOS-Fc 5.0 mg/kg were employed [4,18,34,35]. It is worth noting that the in vivo experiments demonstrated the efficacy of the proposed approach as such (IL MIX), since the administration of free MIX, IL ICOS-Fc, and IL MIX without ICOS-Fc failed to provide substantial therapeutic effects in terms of tumor growth, angiogenesis inhibition, and immunomodulation. The inefficacy of IL ICOS-Fc is in apparent conflict with the previously documented efficacy of ICOS-Fc-loaded PLGA and cyclodextrin nanoparticles in the same tumor model [4]. This discrepancy can be ascribed to the ICOS-Fc dose, which, in IL, was only 25% of that used in the other nanoparticles. However, the effect of ICOS-Fc in the IL MIX is highlighted by the significant loss of the anti-tumor effects displayed in the IL MIX that lacked ICOS-Fc. The main effect of IL MIX appears to be its action against angiogenesis, which may take advantage of the ability of SOR and ICOS-Fc to inhibit VEGF and OPN induction, respectively.

### 4.3. The Role of Immune Modulation

**Figure 9:**
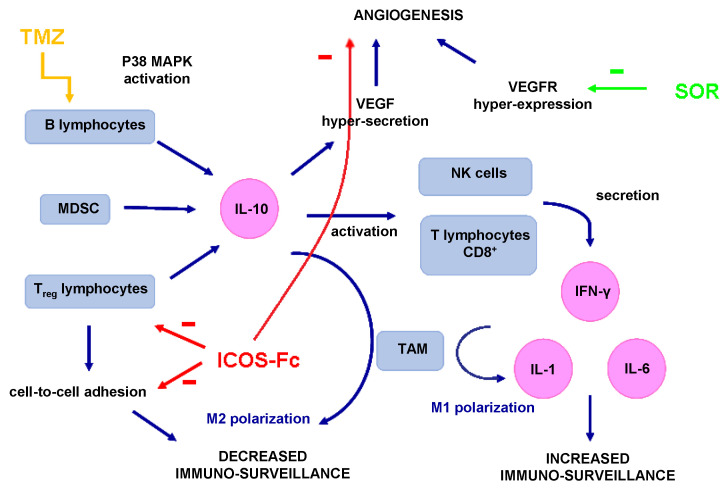
Scheme of immunologic pathways involved in polychemotherapy (MIX). Abbreviations: CD8: cluster of differentiation 8; IFN-γ: interferon-γ; IL-1: interleukin 1; IL-6: interleukin 6; IL-10: interleukin 10; MAPK: mitogen-activated protein kinase; MDSC: myeloid-derived suppressor cells; NK: natural killer; SOR: sorafenib; TAM: tumor-associated macrophages; TMZ: temozolomide; T_reg_: lymphocytes T regulators; VEGF: vascular endothelial growth factor; VEGFR: VEGF receptor.

It is noteworthy that multi-functional nanomedicines able to act as both immunomodulators and drug carriers have been suggested, including autologous microparticles [36]. Within this context, IL MIX also displayed substantial effects in terms of immune modulation, as detected by the increased expression of IFNγ, IL-1, IL-6, and IL-10. Intriguingly, the increase of IL-1, IL-6, and IL-10 was mostly dependent on the whole IL MIX drug combination, since these cytokines were not increased in mice treated with free MIX or IL ICOS and were significantly lower in mice treated with IL MIX without ICOS than in those treated with the whole IL MIX. In contrast, the increase of IFN-γ was independent from the presence of ICOS-Fc, since it was increased at similar levels in mice treated either with IL MIX or IL MIX without ICOS, while it was not increased in mice treated with free MIX or IL ICOS. The increased expression of IL-10 was unexpected since IL-10 usually works as an anti-inflammatory and pro-oncogenic agent because it is associated with regulatory T lymphocytes and the M2 polarization of tumor-associated macrophages. Conversely, IFN-γ is produced by lymphocytes with anti-tumor and pro-inflammatory activity, such as T helper type 1 lymphocytes, cytotoxic T lymphocytes, and natural killer (NK) cells [37]. Moreover, it is well-known that IL promotes the polarization of macrophages to the anti-cancer M1-like phenotype after i.v. administration [33]. However, the effect of IL-10 may vary depending on the tissue context as it can even trigger IFN-γ secretion and increase cytotoxic anti-tumor lymphocytes and tumor rejection [38]. Previous evidence that we have accrued [18,27] suggests that the increase in IL-10 may be mediated by the activation of P38 mitogen-activated protein kinase (MAPK) in B lymphocytes, as induced by cytostatic agents such as TMZ [39,40] (Figure 9). In contrast, the involvement of ICOS-Fc in this mechanism is unlikely, since our previous work has shown that ICOS-Fc decreases IL-10 expression in the tumor mass when loaded into cyclodextrin nanosponges, but not in PLGA nanoparticles. In this scenario, it is also noteworthy that, of the cytokines involved in acute inflammation, the expression of IL-6 and IL-1 was increased, but that of TNF-α was not.

However, the immunological context can vary with the mouse model employed. Indeed, the immunocompetent B16-F10 model in this experimental work was selected because it expresses a large amount of ICOS-L (data not shown). Nonetheless, it is not a BRAF-mutated model. Therefore, we will also assess our approach in BRAF-mutated models in future studies to take advantage of the promising in vitro results obtained in genetically modified BRAF-mutated D4M cells. In this case, selective BRAF inhibitors (such as vemurafenib) could be included in the drug combination to provide the necessary advantages.

## 5. Conclusions

Despite the relevant advances in the pharmacological therapy of high-grade melanoma obtained in recent years, this disease still represents a serious threat. This research work therefore presented a combination therapy that has been engineered to include ICOS-Fc as the immuno-stimulating agent, a cytostatic agent (TMZ), and a kinase inhibitor (SOR) for loading into a nanoemulsion used for total parenteral nutrition. Results showed that this therapy was effective in inhibiting the growth of mouse melanoma in vivo by exerting a potent anti-angiogenic effect and complex immuno-regulatory activity. This is the first attempt to introduce an immunotherapeutic drug working in the ICOS/ICOS-L axis in a polychemotherapy approach, and the results showed that this approach allows to substantially decrease the drug dose needed to obtain a therapeutic effect. Use of ICOS-Fc is innovative since it works as both an immunostimulatory and antiangiogenic agent, and therefore would be optimally synergistic with the other drugs loaded in the nanoparticles. Tumor growth inhibition was obtained without any sign of systemic toxicity. Indeed, sub-therapeutic drug doses were most probably effective because of the hypothesized targeting properties of IL. This approach might represent a potential future tool that can merge immunotherapy, targeted therapy, and chemotherapy into one safe delivery vehicle to improve therapeutic efficacy, without increasing the incidence of the adverse side effects that are typical of combination therapies.

## 6. Patents

PCT IB/2019/050154.

## Figures and Tables

**Table 1:** Characterization of Intralipid^®^ (IL)-based formulations. Abbreviations: EE%: % entrapment efficiency; MIX: drug combination (temozolomide dodecyl ester, sorafenib, ICOS-Fc); N.D.: not determined; PS: polystyrene sulfonate; SOR: sorafenib; TMZ-C12: temozolomide dodecyl ester.

	Mean Size (nm)	Polydispersity	Z Potential (mV)	Recovery %	EE %	
					Size Exclusion	Dextran Gradient
IL MIX (human ICOS-Fc)	279.9 ± 3.0	0.146	−28.50 ± 3.35	TMZ-C12: 94.0 ± 8.0 SOR: 96.1 ± 6.0 ICOS-Fc: 106.3 ± 11.7	TMZ-C12: 95.9 SOR: 84.6	TMZ-C12: 94 SOR: 74
IL MIX (human ICOS-Fc low dose)	269.3 ± 10.1	0.025	−29.41 ± 3.06	TMZ-C12: 100 ± 5.1 SOR: 91 ± 6.7 ICOS-Fc: N.D.	N.D.	N.D.
IL MIX (mouse ICOS-Fc)	270.2 ± 2.1	0.099	−39.08 ± 3.69	TMZ-C12: 99 ± 11.1 SOR: 119 ± 11.8 ICOS Fc: 107 ± 21.4	N.D.	N.D.
IL MIX without ICOS-Fc	257.6 ± 5.0	0.129	−33.58 ± 3.03	TMZ-C12: 91 ± 6.5 SOR: 105 ± 6.0	N.D.	N.D.
IL TMZ-C12	275.0 ± 0.7	0.003	−39.52 ± 7.22	68 ± 5.0	N.D.	N.D.
IL SOR	262.6 ± 1.2	0.071	−20.72 ± 1.91	102.7 ± 11.7	N.D.	N.D.
IL mouse ICOS-Fc	348.3 ± 6.1	0.142	−33.17 ± 4.5	116 ± 10.2	47	97
IL human ICOS-Fc	265.6 ± 1.6	0.028	−26.79 ± 3.40	78 ± 9.8	55.4	97
IL human ICOS-Fc (+PS)	262.0 ± 3.7	0.050	−48.08 ± 8.47	88.7 ± 9.3	N.D.	N.D.
IL human ICOS-Fc low concentration	244.6 ± 18.0	0.051	−15.72 ± 1.83	N.D.	N.D.	N.D.
Blank IL	290.0 ± 1.9	0.005	−39.53 ± 2.07	N.D.	N.D.	N.D.

**Table 2:** Animal experiments (I): organ weights (grams). Abbreviations: CTR: control; IL: Intralipid^®^; MIX: drug combination (temozolomide dodecyl ester, sorafenib, ICOS-Fc).

	Liver	Spleen	Kidneys	Lungs	Heart
**CTR**	1.07 ± 0.06	0.13 ± 0.01	0.25 ± 0.02	0.18 ± 0.03	0.14 ± 0.02
**MIX**	1.05 ± 0.08	0.25 ± 0.04	0.24 ± 0.00	0.15 ± 0.01	0.15 ± 0.03
**IL MIX**	0.91 ± 0.07	0.17 ± 0.05	0.22 ± 0.01	0.16 ± 0.01	0.12 ± 0.01
**IL ICOS-Fc**	1.00 ± 0.06	0.27 ± 0.04	0.22 ± 0.00	0.17 ± 0.01	0.15 ± 0.01

**Table 3:** Animal experiments (II): organ weights (grams). Abbreviations: IL: Intralipid^®^; MIX: drug combination (temozolomide dodecyl ester, sorafenib, ICOS-Fc).

	Liver	Spleen	Kidneys	Lungs	Heart
**IL MIX**	0.86 ± 0.05	0.16 ± 0.04	0.20 ± 0.03	0.28 ± 0.14	0.13 ± 0.00
**IL MIX NO ICOS-Fc**	0.87 ± 0.06	0.18 ± 0.03	0.23 ± 0.01	0.16 ± 0.01	0.23 ± 0.08

## Data Availability

Not applicable.
